# Meteorological factors modulate idiopathic epistaxis risk: a 12-year retrospective study in Changshu

**DOI:** 10.3389/fpubh.2026.1800994

**Published:** 2026-06-30

**Authors:** Chao Yu, Xueting Fu, Lian Gu, Yong Yin, Teng He, Zhen Wu, Hao Wu

**Affiliations:** 1 Medical College of Nantong University, Nantong, Jiangsu, China; 2 Department of Otorhinolaryngology, Changshu No. 1 People’s Hospital, Changshu, Jiangsu, China; 3 Department of Otorhinolaryngology, Changshu No 2 People’s Hospital, Changshu, Jiangsu, China; 4 Department of Otorhinolaryngology Head and Neck Surgery, Nantong First People’s Hospital, Southeast University, Nantong, Jiangsu, China

**Keywords:** epistaxis, humidity, lag effect, meteorological factors, temperature

## Abstract

**Objective:**

To investigate the incidence of idiopathic epistaxis in Changshu, China, and its correlations with meteorological factors.

**Methods:**

We retrospectively enrolled 24,193 patients with idiopathic epistaxis from March 2011 to November 2022. Matched clinical, population, and meteorological data were analyzed. Spearman’s correlation and variance inflation factor analyses were performed to assess multicollinearity. We conducted quartile stratification and sequential robust negative binomial regression with three progressively adjusted models, as well as 1–4 day lag effect analyses.

**Results:**

The highest incidence was in patients <18 years (40.3%) and the lowest in those ≥60 years (14.3%), while males accounted for approximately 60% of all cases. Incidence was higher in spring (32%) and summer (27%). Regression analysis showed that higher relative humidity reduced disease risk by 17% (IRR = 0.83, 95% CI: −21 – −12%, *p* < 0.001). Extremely low atmospheric pressure increased risk by 85% with a threshold effect and no significant linear trend. Higher temperature raised risk by 12% (IRR = 1.12, 95% CI: 0–24%, *p* < 0.05). Wind speed and daily temperature difference showed no significant associations. During lag days 1–4, effects of humidity and daily temperature difference peaked at lag 1 day. Temperature exerted divergent effects at lag 1 and 2 days. No lag effects were observed for wind speed or atmospheric pressure.

**Conclusion:**

Low humidity and high temperature are independent risk factors for idiopathic epistaxis. Atmospheric pressure presents a threshold effect, and meteorological factors exert diverse short-term lag effects on disease onset.

## 1 Introduction

Epistaxis is a significant emergency in the field of otorhinolaryngology and head and neck surgery and is classified as secondary or idiopathic. Secondary epistaxis results from local factors such as infection, chemical irritation, nasal inflammation, allergies, trauma, tumors, and intranasal medications, as well as systemic factors such as anticoagulant use, blood disorders, hypertension, and atherosclerosis, based on their etiology and associated risk factors. In contrast, idiopathic epistaxis typically refers to spontaneous epistaxis without a discernible etiology (1).

There is a recognized relationship between meteorological conditions and the incidence of human illnesses. For example, increases in daily mean temperature and humidity have been associated with an increased likelihood of respiratory diseases, including pneumococcal pneumonia (2); conversely, low temperatures can trigger sympathetic nervous system excitation, potentially leading to cardiovascular and cerebrovascular events (3). Several scholars have investigated the association between environmental meteorological factors and epistaxis incidence, primarily in Republic of Korea (4, 5), the United States (6), Canada (7), Brazil (8), and other regions. However, due to variations in inclusion criteria and research methodologies, the findings are diverse, and nearly all relevant studies have a study duration of less than 5 years. Research on this topic in mainland China is relatively scarce. In the Yangzhou region, only one study spanning 5 years has been conducted (9), whereas most others have focused on the relationship between atmospheric pollutants and epistaxis incidence (10–12).

Accordingly, using 12-year continuous observational data from Changshu, we adopted Spearman correlation analysis and variance inflation factor tests to address multicollinearity among meteorological variables. After stratifying these variables by quartiles, we applied robust negative binomial regression to explore their associations with the incidence of idiopathic epistaxis.

## 2 Materials and methods

### 2.1 Cases and population data of Changshu city

This research defined epistaxis as idiopathic epistaxis, in alignment with the International Classification of Diseases, Tenth Revision (ICD-10) designation R04.0 (1), as diagnosed in the outpatient emergency department. To guarantee diagnostic consistency and rigorous case selection, all cases were screened via uniform and strict inclusion and exclusion criteria.

From March 1, 2011, to November 30, 2022, a total of 30,995 patients with epistaxis were admitted to the outpatient and emergency department of the Second People’s Hospital of Changshu City, Jiangsu Province, China. Patients were screened using the following criteria: (1) onset of epistaxis within 24 h; (2) recurrent epistaxis within 14 days considered a single event; and (3) reside in the Changshu area. The exclusion criteria included (1) history of trauma (including both accidental and surgical trauma); (2) diagnosis of sinusitis or tumors; (3) coagulation disorders (including hematological disorders, liver or kidney dysfunction, and the use of nonsteroidal anti-inflammatory drugs); (4) application of nasal sprays (including those that feature steroids, antihistamines, or ipratropium bromide) for the treatment of rhinitis or allergic rhinitis; and (5) cardiovascular disorders, acute infectious diseases, endocrine disorders, and hereditary hemorrhagic telangiectasia.

**Figure 1:**
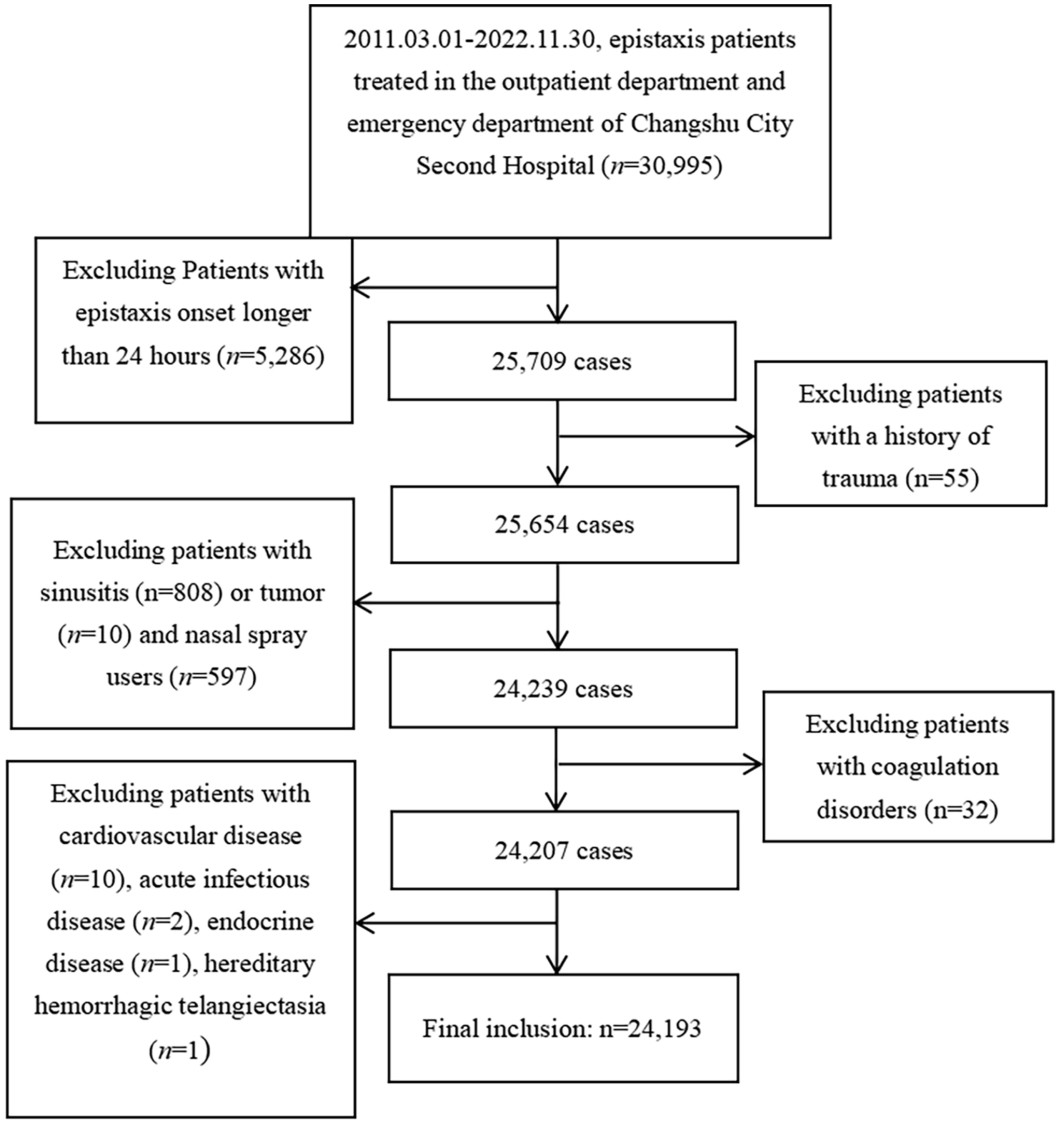
Case screening process.

Ultimately, a total of 24,193 patients were eligible for inclusion, and data on sex, age, and date of onset (the day the patient reported epistaxis) were recorded. The patient inclusion process is shown in Figure 1.

The resident population of Changshu city, including both registered and migrant populations, was categorized by sex and age for each year (<18 years, 18–34 years, 35–59 years, and ≥60 years). Relevant population data were obtained from the official government portal and requests to the appropriate departments.

### 2.2 Meteorological data

Meteorological data from March 1, 2011, to November 30, 2022, including hourly temperature (°C), humidity (%), wind speed (m/s), and atmospheric pressure (hPa) data, were acquired from the ECMWF/NASA historical reanalysis dataset via the Xihe Energy Big Data Platform^1^.

### 2.3 Study data groupings

Patients were categorized based on sex (male and female), age brackets (<18 years, 18–34 years, 35–59 years, and those aged 60 years and above), as well as by season (spring: March to May; summer: June to August; autumn: September to November; and winter: December to February of the subsequent year). In 2022, only three seasons—namely spring, summer, and autumn—were included in the analysis, yielding a total of 2 (sex) × 4 (age groups) × 47 (historical seasons) = 376 subgroups. Daily mean local meteorological data were calculated to obtain the daily mean temperature (mean temperature, MT, °C), daily mean humidity (mean humidity, MH, %), daily mean wind speed (mean wind speed, MWS, m/s), daily mean atmospheric pressure (mean atmospheric pressure, MAP, hPa), and daily temperature difference (mean temperature difference, MTD; also known as diurnal temperature range, DTR, °C). The number of cases was analyzed by subgroup. For each subgroup, the corresponding mean local population and meteorological factors on the day of incidence (MT, MH, MWS, MAP, and MTD) were matched.

### 2.4 Patient characteristics from March 2011 to November 2022

In this study, data from 24,193 patients diagnosed with idiopathic epistaxis were analyzed, focusing on sex, age at onset, age group, season of onset, and year of onset. Age at onset was analyzed as a continuous variable and is reported as the mean ± standard deviation ( $\bar{X}$ ± SD), median (interquartile range, IQR), along with the minimum (Min) and maximum (Max) values. Categorical variables, including sex, age group, season of onset, and year of onset, are reported as frequencies and corresponding constituent ratios.

### 2.5 Spearman’s rank correlation analysis and multicollinearity assessment

Spearman’s rank correlation analysis was performed to examine the correlations between meteorological variables—specifically, mean temperature, humidity, wind speed, atmospheric pressure, and daily temperature difference—from March 2011 to November 2022, so as to preliminarily evaluate the strength of associations among all variables. The variance inflation factor (VIF) was further adopted for multicollinearity testing, to select variables for robust negative binomial regression and eliminate the interference of multicollinearity on model estimation.

### 2.6 Correlation analysis between meteorological factors and idiopathic epistaxis

Each meteorological factor was divided into four quartiles representing different exposure levels (Q1, Q2, Q3, and Q4).

Given evident over-dispersion for subgroup event counts, we chose robust negative binomial regression to analyze how meteorological factors relate to idiopathic epistaxis. Incidence rate ratios (IRRs) and corresponding 95% confidence intervals (CIs) were computed. Definition of incidence rate: This indicator is calculated as the number of new idiopathic epistaxis cases in each subgroup divided by the population size of that subgroup over the same period. In these models, the dependent variable (*Y*) was the number of patients, and the independent variables (*X*) were the meteorological factors. Additionally, adjustments for other variables were considered, and the total population size was included as an offset term. The independent variable was treated as a dummy variable, with Q1 as the reference category. To assess the linear relationship, independent variables were added to the model in increments of 1, 2, 3, and 4, thereby allowing evaluation of how the incidence changed with each added meteorological factor. We utilized a stepwise modeling strategy, building the following models consecutively:

(a) Model 1 was a basic model that does not adjust for any variables.
(b) Model 2 was adjusted only for year of onset, season of onset, patient sex, and patient age at onset.
(c) Model 3 was built on Model 2, with additional meteorological indicators (MT, MH, MWS, MAP, and MTD) incorporated for joint adjustment.

### 2.7 Analysis of lag effects of meteorological factors

We also performed lag analysis covering 1 to 4 days. Using the same robust negative binomial regression model as that for main effect estimation, we fitted the association strength between meteorological indicators and idiopathic epistaxis at each lag day. This part aimed to clarify the delayed onset pattern of the disease following meteorological exposure.

### 2.8 Statistical analysis

Statistical evaluations were conducted utilizing Stata version 18.0 (College Station, Texas 77845 United States). A *p*-value of <0.05 was deemed to represent statistical significance.

Approval for this study was granted by the Ethics Review Committee at the Second People’s Hospital located in Changshu City (Ethics No. 2023-KY-ZX36).

## 3 Results

### 3.1 Demographic characteristics of idiopathic epistaxis patients from 2011 to 2022

**Figure 2:**
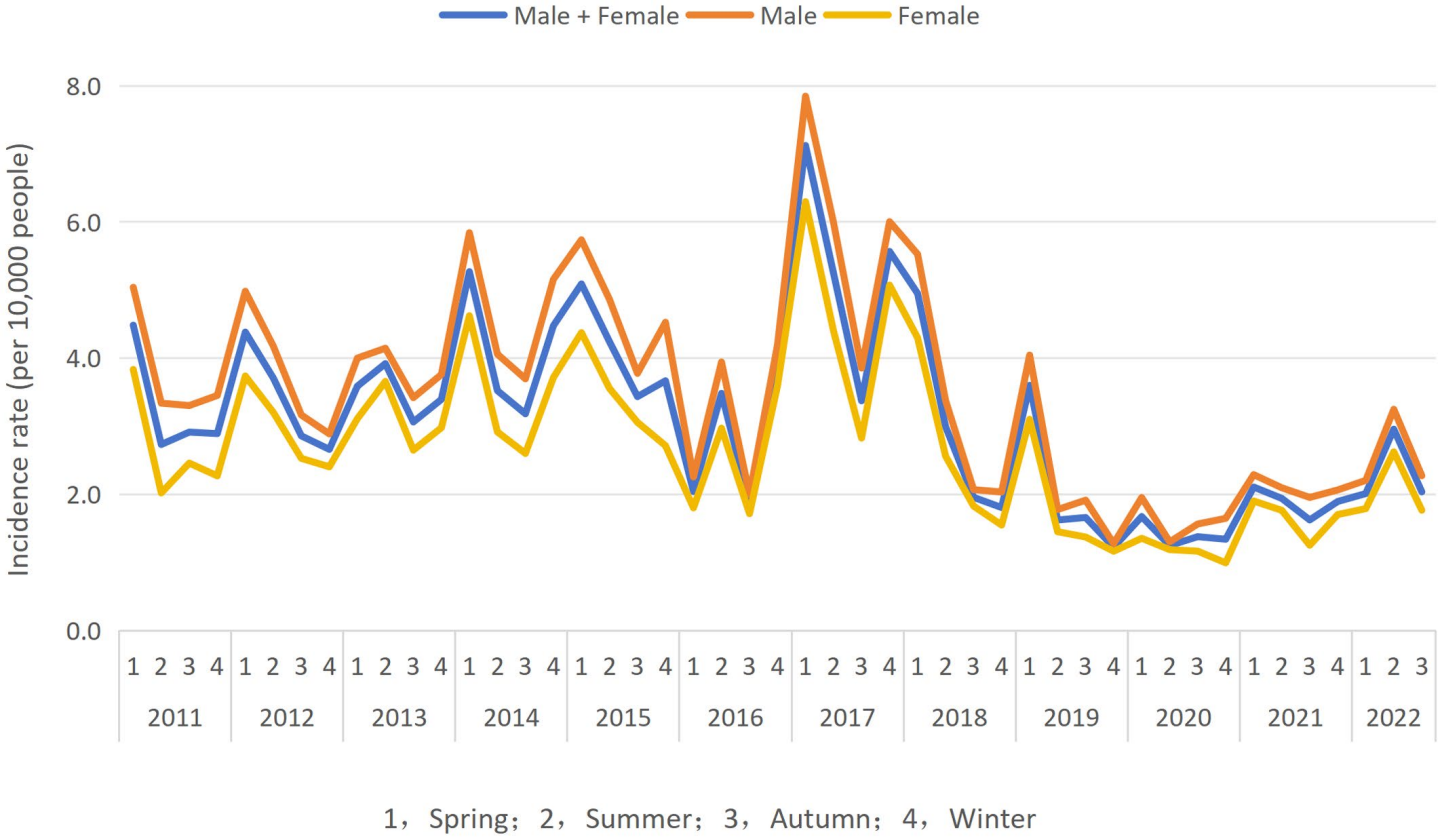
Seasonal and interannual variation trends of idiopathic epistaxis incidence by gender in Changshu region from 2011 to 2022.

(1) The mean age of patients with idiopathic epistaxis was 29.4 years (SD: 22.7 years). The demographic distribution revealed that the lowest proportion of patients (14.3%) was aged 60 years and older, while the highest proportion (40.3%) was under 18 years.
(2) The proportion of males (59.7%) exceeded that of females (40.3%). The mean age at onset was lower in males (27.8 ± 22.3 years) than in females (31.8 ± 23.1 years) (*p* < 0.001).
(3) A significant sex-based difference in age distribution was observed (*p* < 0.001). In the <18 and 18–34 year age groups, the proportion of males (42.7, 25.1%) was greater than that of females (36.6, 21.5%). Conversely, in the 35–59 years and 60 years and above age groups, the proportion of females (25.7 and 16.2%, respectively) exceeded that of males (19.1 and 13.1%, respectively).
(4) No sex difference was observed in the seasonal or annual distribution of disease (p > 0.05) (Table 1).
(5) The seasonal distribution curve revealed a higher number of cases in spring and summer, though the difference was not statistically significant (*p* > 0.05) (Figure 2).

**Table 1:** Demographic characteristics of idiopathic epistaxis patients from 2011 to 2022 (*n* = 24,193).

Characteristics	Overall	Male	Female	*p*-value
Patients, *n* (%)	24,193 (100.0)	14,447 (59.7)	9,746 (40.3)	
Age at onset of symptoms, years				
Mean (SD)	29.4 (22.7)	27.8 (22.3)	31.8 (23.1)	<0.001
Median (Q_1_, Q_3_)	23 (9, 48)	22 (8, 43)	27 (10, 52)	<0.001
Min, Max	0, 97	0, 92	0, 97	
Age group at diagnosis, *n* (%)				<0.001
<18 years	9,741 (40.3)	6,170 (42.7)	3,571 (36.6)	
18~34 years	5,723 (23.7)	3,625 (25.1)	2,098 (21.5)	
35~59 years	5,266 (21.8)	2,764 (19.1)	2,502 (25.7)	
≥60 years	3,463 (14.3)	1,888 (13.1)	1,575 (16.2)	
Season at diagnosis, *n* (%)				0.978
Spring	7,747 (32.0)	4,616 (32.0)	3,131 (32.1)	
Summer	6,522 (27.0)	3,902 (27.0)	2,620 (26.9)	
Autumn	4,861 (20.1)	2,921 (20.2)	1,940 (19.9)	
Winter	5,063 (20.9)	3,008 (20.8)	2,055 (21.1)	
Year at diagnosis, *n* (%)				0.236
2011	2,094 (8.7)	1,302 (9.0)	792 (8.1)	
2012	2,505 (10.4)	1,481 (10.3)	1,024 (10.5)	
2013	2,417 (10.0)	1,411 (9.8)	1,006 (10.3)	
2014	2,735 (11.3)	1,672 (11.6)	1,063 (10.9)	
2015	2,827 (11.7)	1,662 (11.5)	1,165 (12.0)	
2016	1,865 (7.7)	1,140 (7.9)	725 (7.4)	
2017	3,329 (13.8)	1,977 (13.7)	1,352 (13.9)	
2018	2,059 (8.5)	1,214 (8.4)	845 (8.7)	
2019	1,295 (5.4)	761 (5.3)	534 (5.5)	
2020	821 (3.4)	500 (3.5)	321 (3.3)	
2021	1,116 (4.6)	667 (4.6)	449 (4.6)	
2022	1,130 (4.7)	660 (4.6)	470 (4.8)	

*Where appropriate, *p*-values for gender-based differences were calculated using independent samples *t*-tests, Chi-squared tests, or rank-sum tests. Q, quartile; SD, standard deviation; Max, maximum; Min, minimum.

### 3.2 Meteorological data characteristics from March 2011 to November 2022

Meteorological data, including the mean ± standard deviation, median (Q1, Q3), as well as the minimum and maximum values, were analyzed. In addition to humidity, meteorological levels on the day of onset were comparable between male and female patients with epistaxis (*p* > 0.05) (Table 2).

**Table 2:** Meteorological characteristics of idiopathic epistaxis in Changshu, China, March 2011~November 2022.

Meteorological indicators	Overall	Male	Female	*p*-value
Patients, *n* (%)	24,193 (100.0)	14,447 (59.7)	9,746 (40.3)	
MH, %				
Mean (SD)	72.9 (11.4)	73.1 (11.4)	72.7 (11.4)	0.013
Median (Q_1_, Q_3_)	73.5 (65.4, 81.3)	73.8 (65.6, 81.4)	73.3 (65.2, 81.1)	0.017
Min, Max	34.3, 98.1	34.3, 98.1	35.0, 97.9	
MAP, hPa				
Mean (SD)	1015.2 (9.0)	1015.2 (9.0)	1015.2 (9.0)	0.410
Median (Q_1_, Q_3_)	1014.3(1007.4, 1022.3)	1014.3(1007.4, 1022.2)	1014.4(1007.3, 1022.6)	0.540
Min, Max	989.0, 1040.4	989.0, 1040.4	989.0, 1040.4	
MT, °C				
Mean (SD)	18.2 (9.0)	18.3 (9.0)	18.2 (9.1)	0.627
Median (Q_1_, Q_3_)	19.7 (10.3, 25.3)	19.7 (10.6, 25.3)	19.7 (10.1, 25.4)	0.708
Min, Max	−6.8, 36.3	−6.8, 36.3	−6.8, 36.3	
MTD, °C				
Mean (SD)	7.5 (3.2)	7.5 (3.2)	7.5 (3.2)	0.320
Median (Q_1_, Q_3_)	7.3 (5.2, 9.5)	7.3 (5.2, 9.5)	7.3 (5.2, 9.5)	0.365
Min, Max	−0.7, 19.5	−0.7, 19.5	−0.7, 19.5	
MWS, m/s				
Mean (SD)	3.3 (1.1)	3.3 (1.1)	3.2 (1.1)	0.559
Median (Q_1_, Q_3_)	3.1 (2.4, 3.9)	3.1 (2.4, 3.9)	3.1 (2.4, 3.9)	0.597
Min, Max	0.9, 10.6	0.9, 10.6	0.9, 10.6	

*p-values for gender disparities were computed via the independent samples *t*-test, Chi-squared test, or rank-sum test, depending on the statistical context. Q, quartile; SD, standard deviation; Max, maximum; Min, minimum.

### 3.3 Spearman’s rank correlation analysis and multicollinearity assessment

**Figure 3:**
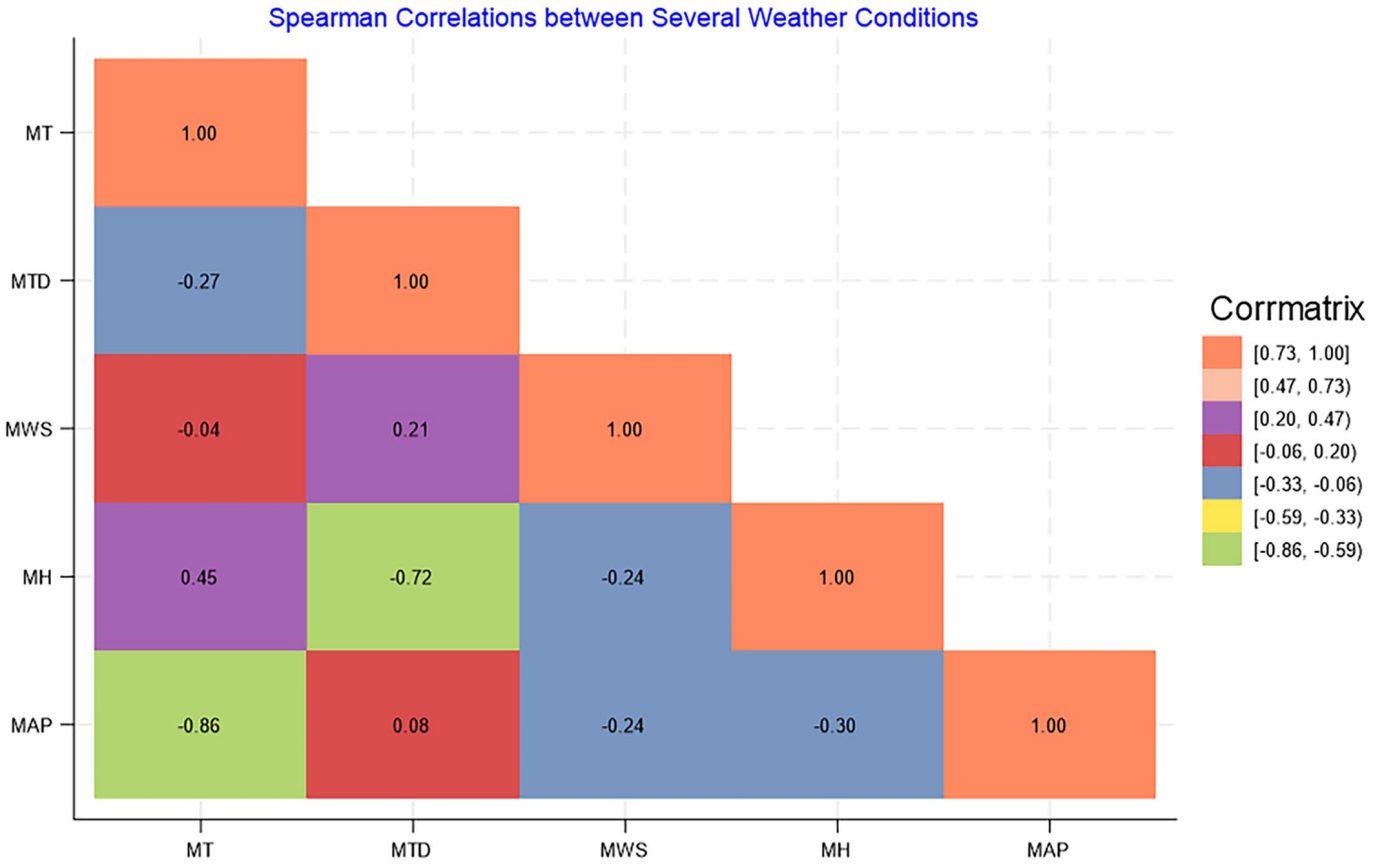
Hierarchical correlation of meteorological factors.

(1) Mean temperature was negatively correlated with the mean atmospheric pressure and daily temperature difference, with correlation coefficients of −0.86 and −0.27. Conversely, it was positively correlated with mean humidity (*r* = 0.45). Additionally, the daily temperature difference was negatively correlated with mean humidity (*r* = −0.72) and positively correlated with mean wind speed (*r* = 0.21). Furthermore, mean wind speed was negatively correlated (*r* = −0.24) with both mean humidity and mean atmospheric pressure. Finally, mean humidity was negatively correlated with mean atmospheric pressure (*r* = −0.30). No significant correlations were observed among the other indicators (Figure 3).
(2) VIF was used to assess multicollinearity. The VIF values of all included variables were <10, with the maximum value reaching 8.06 and the mean value being merely 2.87. These results indicated no severe multicollinearity among variables (Table 3).

**Table 3:** Multicollinearity test results of study variables by VIF.

Variable	VIF	1/VIF
MAP	8.06	0.124069
MT	5.61	0.178242
Season	3.15	0.317210
MH	1.63	0.614745
MWS	1.37	0.730086
Year	1.08	0.925787
Age	1.06	0.941087
Male	1.01	0.993234
Mean VIF	2.87	—

VIF refers to variance inflation factor. VIF < 10 indicates no severe multicollinearity.

### 3.4 Correlation between meteorological factors and idiopathic epistaxis

#### 3.4.1 Relationship between mean humidity and idiopathic epistaxis

Patients were categorized based on mean humidity quartiles, from lowest to highest, with the daily mean humidity for the four quartiles recorded at 67.7, 72.5, 75.8, and 79.9%, respectively (Table 4).

**Table 4:** Associations [IRR (95% CI)] between MH and risk of idiopathic epistaxis^a^.

Indicators	Quartiles of MH				MH^b^	
	Q1 (lowest)	Q2	Q3	Q4 (highest)	↑1 level	*p*_trend_
MH, %						
Mean	67.7	72.5	75.8	79.9	─	─
Min, Max	56.9, 70.4	70.5, 74.1	74.2, 77.3	77.3, 85.6	─	─
No. of event	8,349	5,688	5,678	4,478	─	─
Model 1^c^	1.76 (1.25, 2.48)	1.08 (0.78, 1.49)	1.30 (0.89, 1.89)	1 (ref.)	0.86 (0.77, 0.95)	0.005
Model 2^d^	0.85 (0.74, 0.96)	0.74 (0.63, 0.87)	0.60 (0.51, 0.71)	1 (ref.)	0.84 (0.80, 0.89)	<0.001
Model 3^e^	1.73 (1.45, 2.05)	1.45 (1.24, 1.69)	1.25 (1.11, 1.41)	1 (ref.)	0.83 (0.79, 0.88)	<0.001

MH, mean humidity; Min, minimum; Max, maximum.

a Robust negative binomial regression was used to examine the correlation between MH and idiopathic epistaxis incidence, with the patient count as the dependent variable and the matched population size serving as the offset.

b As a continuous variable, linear trends for MH across quartiles (Q1–Q4) were assessed by assigning ordinal values of 1 to 4.

c Model 1: crude model without adjustment for confounding factors.

d Model 2: adjustments were made for sex at diagnosis, age group (<18 years, 18–34 years, 35–59 years, and ≥60 years), as well as diagnosis year and season.

e Model 3: based on Model 2, mean temperature, wind speed and atmospheric pressure were further adjusted (all variables were incorporated into the model as dummy variables categorized by quartiles).

Compared with patients in the highest mean humidity quartile (Q4), the risk of idiopathic epistaxis rose progressively as mean humidity decreased. Model 3 (the final model) indicated that, compared with patients in Q4, the incremental risk of idiopathic epistaxis for patients in Q3, Q2, and Q1 was 25% (95% CI: 11–41%), 45% (95% CI: 24–69%), and 73% (95% CI: 45–105%), respectively.

Furthermore, the data revealed a distinct linear trend, whereby each one-quartile increase in mean humidity corresponded to a mean 17% (95% CI: −21 – −12%) reduction in the risk of idiopathic epistaxis (*p* < 0.001).

#### 3.4.2 Correlation between mean atmospheric pressure and idiopathic epistaxis

Patients were categorized based on mean atmospheric pressure quartiles, ranging from lowest to highest. The daily mean atmospheric pressures for the four quartiles were 1005.2 hPa, 1014.5 hPa, 1018.1 hPa, and 1026.1 hPa (Table 5).

**Table 5:** Relationships [IRR (95% CI)] between MAP and risk of idiopathic epistaxis^a^.

Indicators	Quartiles of MAP				MAP^b^	
	Q1 (lowest)	Q2	Q3	Q4 (highest)	↑1 level	*p*_trend_
MAP, hPa						
Mean	1005.2	1014.5	1018.1	1026.1	─	─
Min, Max	1003.2, 1006.8	1007.2, 1016.5	1016.5, 1020.0	1020.1, 1028.8	─	─
No. of event	6,487	7,737	4,775	5,194	─	─
Model 1^c^	1.67 (1.22, 2.28)	1.91 (1.51, 2.42)	0.93 (0.73, 1.18)	1 (ref.)	0.80 (0.73, 0.87)	<0.001
Model 2^d^	1.79 (0.94, 3.41)	0.89 (0.66, 1.20)	0.91 (0.70, 1.18)	1 (ref.)	1.01 (0.90, 1.13)	0.911
Model 3^e^	1.85 (1.00, 3.41)	0.91 (0.66, 1.24)	0.90 (0.67, 1.21)	1 (ref.)	0.98 (0.88, 1.10)	0.767

MAP, mean atmospheric pressure; Min, minimum; Max, maximum.

a Robust negative binomial regression was applied to quantify the association between MAP and idiopathic epistaxis risk, taking the number of patients as the dependent variable and using the matched population size as the offset term.

b MAP was treated as a continuous factor, and linear trends across Q1–Q4 were validated using sequential values of 1 through 4.

c Model 1: unadjusted baseline model.

d Model 2: models were adjusted for sex at diagnosis, age group (<18 years, 18–34 years, 35–59 years, ≥60 years), year of diagnosis, and season of diagnosis.

e Model 3: on the basis of Model 2, mean temperature, humidity, and wind speed were further adjusted (each variable was entered into the model as a dummy variable, stratified by quartile groups).

Compared with patients in the highest mean atmospheric pressure quartile (Q4), the risk of idiopathic epistaxis initially did not change significantly with decreasing mean pressure. However, when the pressure decreased below a certain threshold, the incidence risk markedly increased, demonstrating a clear “threshold” phenomenon. Model 3 (the final model) indicated that, compared with that in Q4, the incidence risk in Q3 and Q2 did not increase. In contrast, Q1 exhibited a significant 85% increase in incidence risk (95% CI: 0–241%). Because of the threshold phenomenon, linear trend analysis failed to reveal an association between the two variables (*p* = 0.767).

#### 3.4.3 Correlation between mean wind speed and idiopathic epistaxis

Patients were assigned to groups (Q1, Q2, Q3, and Q4) based on mean wind speed quartiles, with mean wind speeds of 2.8 m/s, 3.1 m/s, 3.3 m/s, and 3.6 m/s, respectively (Table 6).

**Table 6:** Correlations [IRR (95% CI)] between MWS and risk of idiopathic epistaxis^a^.

Indicators	Quartiles of MWS				MWS^**b**^	
	Q1 (lowest)	Q2	Q3	Q4 (highest)	↑1 level	*p*_trend_
MWS, m/s						
Mean	2.8	3.1	3.3	3.6	─	─
Min, Max	2.4, 3.0	3.0, 3.2	3.2, 3.4	3.4, 4.1	─	─
No. of event	4,880	5,606	6,331	7,376	─	─
Model 1^c^	1 (ref.)	1.07 (0.74, 1.55)	1.26 (0.89, 1.80)	1.48 (1.02, 2.14)	1.14 (1.02, 1.28)	0.023
Model 2^d^	1 (ref.)	0.94 (0.82, 1.07)	1.05 (0.92, 1.19)	1.02 (0.87, 1.20)	1.02 (0.97, 1.07)	0.433
Model 3^e^	1 (ref.)	0.96 (0.84, 1.08)	1.11 (0.98, 1.26)	1.06 (0.91, 1.24)	1.04 (0.99, 1.09)	0.126

MWS, mean wind speed; Min, minimum; Max, maximum.

a Robust negative binomial regression analyses were performed to explore the association between MWS and idiopathic epistaxis occurrence, where the number of patients was set as the dependent variable, and the size of the matched population was designated as the offset.

b MWS served as a continuous variable, and linear trends for Q1–Q4 were evaluated using assigned values of 1 to 4.

c Model 1: baseline model with no covariates adjusted.

d Model 2: covariates included sex at diagnosis, age stratification (<18 years, 18–34 years, 35–59 years, and ≥60 years), diagnosis year, and diagnosis season.

e Model 3: based on Model 2, mean temperature, humidity, and atmospheric pressure were further adjusted (all factors were included in the model as dummy variables, with classification based on quartiles).

In comparison to patients subjected to the lowest mean wind speed levels, those in Q3 and Q4 demonstrated a heightened risk of idiopathic epistaxis as mean wind speed rose across all three models. In Model 3 (the final model), there was a 11% (95% CI: −2–26%) and a 6% (95% CI: −9–24%) increase in idiopathic epistaxis risk for Q3 and Q4, respectively, compared with exposure to the lowest mean wind speed level. In contrast, there was a 4% decrease (95% CI: −16 – 8%) in idiopathic epistaxis risk in Q2. Furthermore, each one-quartile increase in mean wind speed was correlated with a 4% increase in idiopathic epistaxis risk (95% CI: -1–9%) (*p* = 0.126).

#### 3.4.4 Correlation between mean temperature and idiopathic epistaxis

Patients were categorized into four groups (Q1, Q2, Q3, and Q4) based on mean temperature quartiles, and the mean temperatures in the four quartiles were 6.4 °C, 16.6 °C, 19.8 °C, and 28.0 °C (Table 7).

**Table 7:** Links [IRR (95% CI)] between MT and risk of idiopathic epistaxis^a^.

Indicators	Quartiles of MT				MT^**b**^	
	Q1 (lowest)	Q2	Q3	Q4 (highest)	↑1 level	*p*_trend_
MT, °C						
Mean	6.4	16.6	19.8	28.0	─	─
Min, Max	3.9, 14.2	14.4, 18.3	18.3, 25.3	25.3, 30.8	─	─
No. of event	5,470	5,532	6,740	6,451	─	─
Model 1^c^	1 (ref.)	0.86 (0.70, 1.06)	1.74 (1.36, 2.22)	1.56 (1.15, 2.12)	1.23 (1.12, 1.34)	<0.001
Model 2^d^	1 (ref.)	0.93 (0.77, 1.12)	1.03 (0.81, 1.30)	1.64 (0.80, 3.37)	1.08 (0.96, 1.22)	0.179
Model 3^e^	1 (ref.)	0.95 (0.81, 1.13)	1.09 (0.87, 1.35)	1.50 (0.79, 2.84)	1.12 (1.00, 1.24)	0.047

MT, mean temperature; Min, minimum; Max, maximum.

a Robust negative binomial regression was utilized to analyze the relationship between MT and idiopathic epistaxis incidence, with the total number of patients as the dependent variable and the matched population volume employed as the offset.

b For MT (treated as a continuous variable), linear trends among Q1–Q4 quartiles were tested via ordinal assignment of values 1 to 4.

c Model 1: no adjustment for any covariates.

d Model 2: analyses were adjusted for sex at diagnosis, age group (<18, 18–34, 35–59, and ≥60 years), along with the year and season of diagnosis.

e Model 3: on the basis of Model 2, mean humidity, wind speed, and atmospheric pressure were further adjusted (these variables were introduced into the model as dummy variables classified according to quartiles).

Q3 and Q4 exhibited a heightened risk of idiopathic epistaxis across all three models as the mean air temperature increased relative to patients exposed to the lowest mean air temperature. In Model 3 (the final model), there was a 9% (95% CI: −13–35%) increase in the incidence of idiopathic epistaxis in Q3 and a 50% (95% CI: −21–184%) increase in Q4 relative to exposure to the lowest mean temperature. Conversely, Q2 exhibited a 5% decrease in idiopathic epistaxis incidence (95% CI: −19–13%). Furthermore, the incidence of idiopathic epistaxis increased by 12% (95% CI: 0–24%) for each one-quartile increase in mean air temperature (*p* = 0.047).

#### 3.4.5 Correlation between daily temperature difference and idiopathic epistaxis

Patients were categorized into groups (Q1, Q2, Q3, and Q4) based on daily temperature difference quartiles. The mean daily temperature range levels for the four groups were 5.9 °C, 6.8 °C, 7.6 °C, and 9.1 °C (Table 8).

**Table 8:** Associations [IRR (95% CI)] between MTD and risk of idiopathic epistaxis^a^.

Indicators	Quartiles of MTD				MTD^b^	
	Q1 (lowest)	Q2	Q3	Q4 (highest)	↑1 level	*p*_trend_
MTD, °C						
Mean	5.9	6.8	7.6	9.1	─	─
Min, Max	4.4, 6.5	6.5, 7.2	7.2, 8.0	8.0, 10.9	─	─
No. of event	5,563	5,283	6,469	6,878	─	─
Model 1^c^	1 (ref.)	0.93 (0.65, 1.33)	1.04 (0.73, 1.48)	1.15 (0.82, 1.62)	1.05 (0.95, 1.17)	0.330
Model 2^d^	1 (ref.)	1.20 (1.06, 1.36)	1.26 (1.09, 1.46)	1.38 (1.15, 1.66)	1.11 (1.05, 1.19)	0.001
Model 3^e^	1 (ref.)	1.06 (0.93, 1.20)	1.09 (0.92, 1.30)	1.11 (0.89, 1.37)	1.04 (0.97, 1.11)	0.316

MTD, mean temperature difference; Min, minimum; Max, maximum.

a Robust negative binomial regression analysis was utilized to explore the relationship between MTD and the risk of idiopathic epistaxis, using the patient number as the dependent variable, with the matched population size incorporated as the offset.

b MTD was considered a continuous variable, and linear trends for Q1–Q4 were assessed using sequential numerical values of 1 to 4.

c Model 1: unadjusted model.

d Model 2: statistical adjustment accounted for sex at diagnosis, age categories (<18 years, 18–34 years, 35–59 years, and ≥60 years), as well as diagnosis year and season.

e Model 3: on the basis of Model 2, mean humidity, wind speed and atmospheric pressure were further adjusted (all indicators were input into the model as dummy variables grouped by quartiles).

Q3 and Q4 exhibited an increased risk of idiopathic epistaxis across all two models as the daily temperature difference increased, in comparison with patients subjected to the smallest daily temperature fluctuations. The final model (Model 3) indicated increases in idiopathic epistaxis risk of 6% (95% CI: −7–20%), 9% (95% CI: −8–30%), and 11% (95% CI: −11–37%) in Q2, Q3, and Q4, respectively, relative to exposure to the lowest daily temperature difference. Furthermore, each increase in the daily temperature difference one-quartile was linked to a 4% (95% CI: −3–11%) increase in idiopathic epistaxis risk (*p* = 0.316).

#### 3.4.6 Summary of Incidence Rate Ratios of Idiopathic Epistaxis by Quartiles of Meteorological Factors (Fully Adjusted Model 3)

After mutual adjustment for multiple factors, we summarized the association effects between quartile levels of each meteorological factor and the incidence of idiopathic epistaxis (Table 9).

**Table 9:** Summary of incidence rate ratios of idiopathic epistaxis according to quartiles of meteorological factors (Model 3, final adjusted model).

Meteorological factors	Q1 (lowest)	Q2	Q3	Q4 (highest)	IRR (95% CI) per 1 level change	*p*_trend_
MH, %	1.73 (1.45, 2.05)	1.45 (1.24, 1.69)	1.25 (1.11, 1.41)	1 (ref.)	↑ 1 level: 0.83(0.79, 0.88)	<0.001
MAP, hPa	1.85 (1.00, 3.41)	0.91 (0.66, 1.24)	0.90 (0.67, 1.21)	1 (ref.)	↑ 1 level: 0.98(0.88, 1.10)	0.767
MWS, m/s	1 (ref.)	0.96 (0.84, 1.08)	1.11 (0.98, 1.26)	1.06 (0.91, 1.24)	↑ 1 level: 1.04(0.99, 1.09)	0.126
MT, °C	1 (ref.)	0.95 (0.81, 1.13)	1.09 (0.87, 1.35)	1.50 (0.79, 2.84)	↑ 1 level: 1.12(1.00, 1.24)	0.047
MTD, °C	1 (ref.)	1.06 (0.93, 1.20)	1.09 (0.92, 1.30)	1.11 (0.89, 1.37)	↑ 1 level: 1.04(0.97, 1.11)	0.316

Q1–Q4 represent quartile levels from the lowest to the highest. Reference group: Q4 (highest) for MH and MAP; Q1 (lowest) for MWS, MT, and MTD. IRR, incidence rate ratio; CI, confidence interval; MH, mean humidity; MAP, mean atmospheric pressure; MWS, mean wind speed; MT, mean temperature; MTD, mean temperature difference.

### 3.5 Analysis of lag effects of meteorological factors on idiopathic epistaxis onset

**Figure 4:**
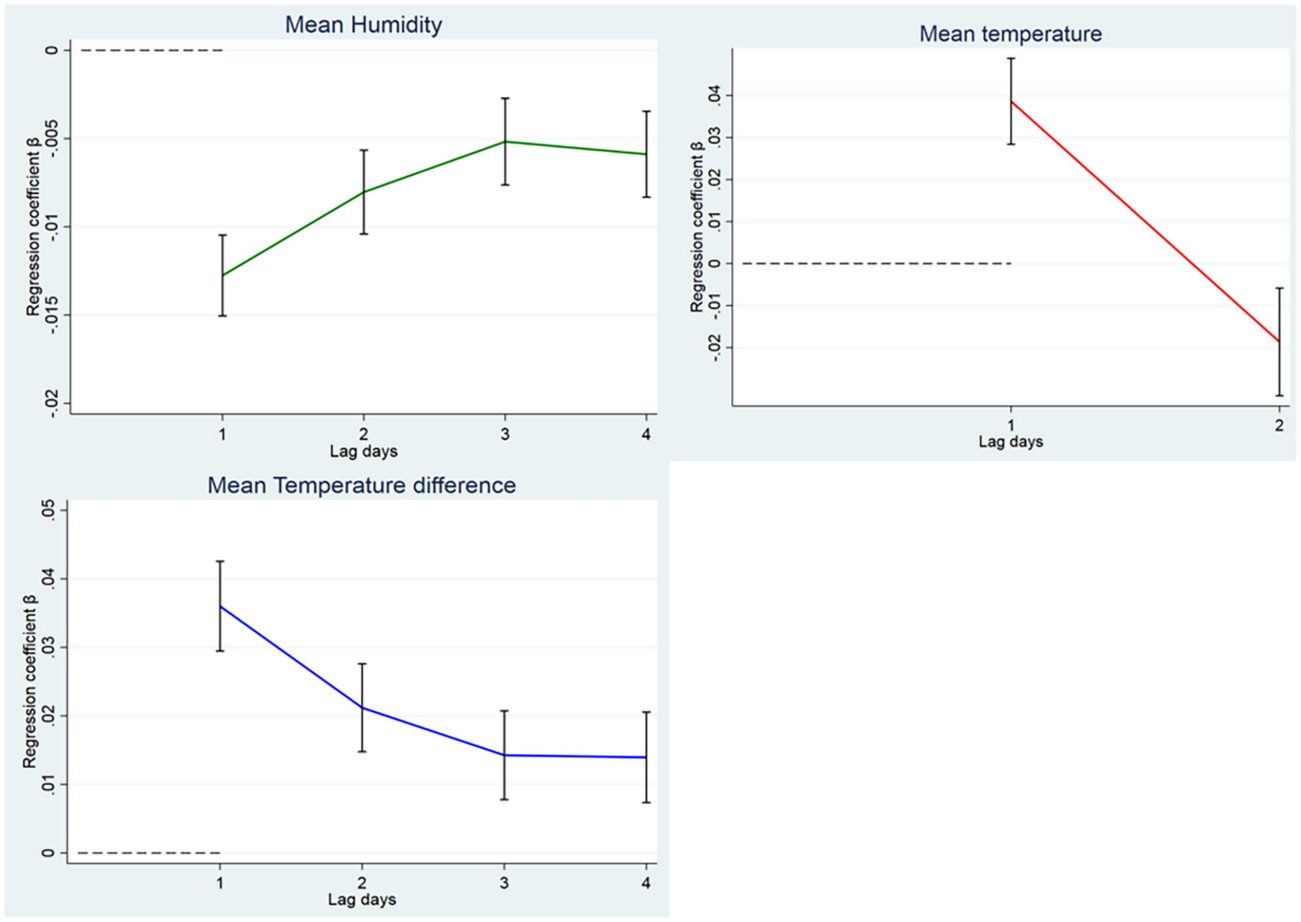
Analysis of the lag effects of various meteorological factors on the onset of idiopathic epistaxis.

A continuous negative lag association was found for mean humidity, with significant results seen from lag day 1 to lag day 4. The magnitude of this effect was greatest on the first lag day and decreased progressively with longer lag periods. The impact of mean temperature shifted direction over time: it was linked to elevated disease risk at lag day 1, and produced a protective effect at lag day 2, both of which were statistically significant. Mean temperature difference was associated with a steady positive lag effect across lag days 1–4, with the strongest effect occurring at lag day 1 followed by a gradual reduction. By contrast, mean wind speed and mean atmospheric pressure did not present notable lag effects (Figure 4).

## 4 Discussion

A suitable environment is a prerequisite for human survival and health. Meteorological factors are a significant component of environmental influences and primarily include temperature, air pressure, wind direction, wind speed, humidity, and their derivative variables, such as temperature differences and air pressure variations. Specific meteorological conditions have been closely linked to the occurrence and progression of many human diseases (2, 3).

As a prevalent disorder in otolaryngology, epistaxis is frequently encountered in clinical practice. Based on data from Changshu, Jiangsu, China, our results indicated an inverse relationship between mean humidity and idiopathic epistaxis, and a positive relationship for mean temperature. For mean atmospheric pressure, an increased disease risk was only identified at extremely low levels, suggesting a threshold effect rather than an overall linear trend. Neither mean wind speed nor daily temperature difference was independently correlated with idiopathic epistaxis onset. Lag analysis further confirmed that the influences of meteorological factors were not restricted to same-day exposure and were strongly time-dependent in the short term. Mean humidity provided persistent protection over multiple lag days, mean temperature had a transient bidirectional effect, and daily temperature difference led to delayed risk. No evident lag associations were detected for mean wind speed and mean atmospheric pressure.

The vast and convoluted nasal mucosa, along with its rich vascular network and glands, regulates the temperature and humidity of the inhaled air to levels suitable for the human body. Damage to this vascular network often leads to nasal bleeding. In dry external environments, the likelihood of damage to the submucosal blood vessels increases, thereby raising the risk of nasal bleeding (4, 7, 8). Our results demonstrated that a one-quartile rise in mean humidity reduced idiopathic epistaxis incidence by 17%, further supporting the above conclusion. Lag effect analysis indicated that nasal mucosal injury caused by low humidity is not immediate, but accumulates and persists for 1 to 4 days. This trend accords well with the short-term lag protection of humidity seen in respiratory illnesses, and explains why epistaxis cases stay high in the days following dry conditions (13). Furthermore, mean atmospheric pressure had no overall negative association with idiopathic epistaxis. The elevated risk solely detected in the lowest atmospheric pressure quartile reflected a threshold effect, which was basically consistent with the results reported by Kwak et al. (5). Some studies have shown that low atmospheric pressure increases the risk of cerebral hemorrhage, as another hemorrhagic condition. These authors suggest that lowered atmospheric pressure may raise bleeding risk in hypertensive patients with arteriosclerosis by reducing vascular reactivity (14, 15). Another study reported that low atmospheric pressure increases the risk of postoperative bleeding following adenoidectomy in children (16), although the underlying mechanisms require further investigation.

No significant independent association was observed between mean wind speed and epistaxis occurrence (*p* = 0.126). A recent large-scale meta-analysis likewise reported no notable independent relationship between monthly mean wind speed and epistaxis prevalence (17). Theoretically, high wind speeds may promote nasal cavity dryness, thereby increasing the risk of vascular damage to the nasal mucosa (18). Some researchers have also suggested that increased concentrations of suspended particles during high wind conditions irritate the nasal mucosa, leading to a higher incidence of rhinitis and indirectly promoting epistaxis (4, 19). After mutual adjustment for multiple meteorological factors, we found that wind speed had no independent pathogenic effect under the confounding and interactions of temperature, humidity, atmospheric pressure and other meteorological variables. This further indicates the complexity of interactions among meteorological factors.

Temperature, a critical environmental factor, has been the subject of numerous studies examining its association with epistaxis, yielding complex results. In this study, which included participants across all age groups, we found that mean temperature in the Changshu area was positively correlated with epistaxis. Furthermore, mean temperature presented distinct time-varying effects. It was linked to elevated risk on lag day 1 and protective effects on lag day 2, demonstrating short-term bidirectional characteristics with strong time dependence. Following multivariate adjustment, no independent correlation was identified between daily temperature difference and epistaxis incidence (*p* = 0.316). Nevertheless, persistent positive lag effects were observed for 1 to 4 consecutive days. This indicates that the pathogenic effect of daily temperature difference is delayed, as it results from repeated vascular changes and cumulative injury to the nasal mucosa. Accordingly, the lack of significance in the concurrent model is reasonable, and the above results are consistent with each other. In contrast, the Brazilian scholar Mangussi-Gomes reported a negative correlation between mean temperature and the incidence of epistaxis in a study involving individuals of all age groups (8). Among studies focusing on children younger than 18, it has been consistently reported that higher mean temperatures correlate with an increased probability of epistaxis (9, 10, 20, 21). In terms of physiological mechanisms, an increase in temperature can lead to nasal vasodilation, and a significant temperature fluctuation, on the one hand, can lead to strong contraction and relaxation of nasal mucosal blood vessels (22); on the other hand, it can lead to nasal mucosal dysfunction, and the ciliary oscillation frequency decreases or even stops, resulting in dry nasal mucosa and scab (6). Despite the evident physiological basis and delayed pathogenic effects of temperature difference, no independent association was observed in the fully adjusted concurrent model, owing to mutual confounding and synergistic effects among meteorological factors. Such inconsistent findings are frequently reported in climate-related health research. Curiously, Zhu et al. (9) reported a negative correlation between temperature difference and the incidence of epistaxis; however, the author did not further discuss these findings. This also implies that the associations between temperature difference and epistaxis differ considerably across regions, study populations and analytical strategies.

In fact, we believe that the correlation between temperature and its derived indicators, such as temperature difference, maximum temperature, minimum temperature, and the incidence of epistaxis, requires comprehensive consideration of multiple factors. American scholar Wei et al. (6) divided the United States into eight regions based on mean temperature and found that extremely hot and extremely cold areas had a higher epistaxis incidence than temperate regions. Furthermore, some researchers have reported that lower minimum temperatures were linked to a higher incidence of epistaxis, attributing this largely to indoor dryness caused by heating under low-temperature conditions rather than the temperature itself (4, 23). Moreover, even in regions with similar latitudes and mean temperatures, inconsistent findings have emerged due to differences in climate types (9, 18, 20, 23).

In summary, the effects of meteorological factors on the incidence of epistaxis are highly complex, as various factors, including the study population, age groups, research regions, and methodologies, may influence the results. In addition to immediate correlations, meteorological factors exert lag effects of different time spans on epistaxis. The lag patterns of mean humidity, mean temperature, and diurnal temperature difference are consistent with those reported for respiratory diseases. This supports that nasal mucosa responds to meteorological exposure in a time-cumulative and delayed manner, complementing the chronological evidence for the association between meteorological conditions and epistaxis. For instance, some scholars believe that meteorological factors have a greater effect on children than on adults due to children’s school attendance and increased outdoor activities (20). Based on a long-term and large sample of 24,193 idiopathic epistaxis cases from Changshu, the present study controlled for mutual confounding and interactive effects across meteorological variables. The additional lag effect analysis further helped approximate real-world meteorological exposure scenarios.

The present study provides epidemiological evidence for regional public health prevention and control of idiopathic epistaxis in Changshu, China. Based on immediate associations and lag effect characteristics, low humidity, extremely low atmospheric pressure, and high temperature can be regarded as core meteorological warning indicators for an increased risk of idiopathic epistaxis.

Several limitations of this study should be noted. First, meteorological exposure was estimated based on regional monitoring station data, which only reflects population average exposure levels and fails to precisely characterize individual personal exposure. Unaccounted differences in indoor microenvironments, occupational exposure conditions, and individual outdoor activity time may introduce potential exposure measurement bias. Second, mild hypertension, undocumented medication use, and minor trauma were incompletely documented due to the retrospective nature of this study. Third, this study did not include atmospheric pollutants such as PM2.5 and PM10, which may act as potential confounders for idiopathic epistaxis. Fourth, this was a single-center study, which may introduce selection and geographic bias and limit the generalizability of our results. Future studies should integrate meteorological and air pollution data, improve individual exposure assessment, and comprehensively collect comorbidities and medication information, conduct multi-center and population-based investigations, and further adjust for confounders and enhance the generalizability and causal inference of our findings.

## 5 Conclusion

Low humidity and high temperature independently increase the risk of idiopathic epistaxis. Atmospheric pressure shows a threshold effect, and different meteorological factors generate varied short-term lag effects on disease onset.

## Data Availability

The raw clinical datasets generated and analyzed in this study contain protected health information that cannot be fully deposited publicly to safeguard patient confidentiality under local medical ethics requirements. De-identified anonymized data supporting the conclusions will be shared with qualified investigators upon reasonable written request to the corresponding author, without undue reservation.

## Footnotes

1
https://xihe-energy.com/
